# Advanced uracil DNA glycosylase-supplemented real-time reverse transcription loop-mediated isothermal amplification (UDG-rRT-LAMP) method for universal and specific detection of Tembusu virus

**DOI:** 10.1038/srep27605

**Published:** 2016-06-07

**Authors:** Yi Tang, Hao Chen, Youxiang Diao

**Affiliations:** 1 College of Animal Science and Veterinary Medicine, Shandong Agricultural University, #61 Dai Zong Avenue Tai’an, Shandong 271018, China

## Abstract

Tembusu virus (TMUV) is a mosquito-borne flavivirus which threatens both poultry production and public health. In this study we developed a complete open reading frame alignment-based rRT-LAMP method for the universal detection of TUMV. To prevent false-positive results, the reaction was supplemented with uracil DNA glycosylase (UDG) to eliminate carryover contamination. The detection limit of the newly developed UDG-rRT-LAMP for TMUV was as low as 100 copies/reaction of viral RNA and 1 × 10^0.89^ − 1 × 10^1.55^ tissue culture infectious dose/100 μL of viruses. There were no cross-reactions with other viruses, and the reproducibility of the assay was confirmed by intra- and inter-assay tests with variability ranging from 0.22–3.33%. The new UDG-rRT-LAMP method for TMUV produced the same results as viral isolation combined with RT-PCR as the “gold standard” in 96.88% of cases for 81 clinical samples from subjects with suspected TMUV infection. The addition of UDG can eliminate as much as 1 × 10^−16^ g/reaction of contaminants, which can significantly reduce the likelihood of false-positive results during the rRT-LAMP reaction. Our result indicated that our UDG-rRT-LAMP is a rapid, sensitive, specific, and reliable method that can effectively prevent carryover contamination in the detection of TMUV.

Tembusu virus (TUMV) belongs to the Ntaya virus group in the genus *Flavivirus* and family *Flaviviridae*^1^. The TMUV particle is approximately 50–60 nm in diameter and a positive-sense, single-stranded RNA genome is packaged by the capsid protein into a nucleocapsid surrounded by a lipid envelope from host cells^2^,^3^. The 5′ capped TMUV genome is approximately 11 kb in length and has one open reading frame (ORF) encoding a single 3410-amino acid residue polyprotein. ORF is flanked by approximately 145- nucleotide (nt) 5′ and 618-nt 3′ non-translated regions^4^,^5^. The N-terminus of the genome encodes three structural proteins, namely capsid, envelope (E), and precursor of membrane. Seven non-structural (NS) proteins, NS1, NS2A, NS2B, NS3, NS4A, NS4B, and NS5^4^, which are essential for viral replication, are encoded by the remainder of the genome^6^. Among these proteins, NS5 is the largest and most conserved protein and it plays essential roles in viral genome capping and replication processes through its N-terminal methyltransferase domain and C-terminal RNA-dependent RNA polymerase (RdRP) domain, respectively. NS5 has been popular for the universal detection of many other flaviviruses^7^,^8^.

TMUV was first reported and named during an arbovirus surveillance study in the 1970s in Malaysia, and the main virus vector was found to be *Culex* mosquitoes^9^. Humans and birds were considered to be infrequently infected with TMUV at that time^10^. Subsequently, a TMUV strain (designated as Sitiawan virus) was isolated from sick broiler chicks in chicken embryos, and it was found to cause encephalitis, growth retardation, and increased blood glucose levels in infected chickens^11^. Since 2010, a highly contagious disease has spread throughout most breeder, layer, and meat-type duck farms in China, causing significant economic losses in the Chinese duck industry^2^. In addition to ducks, TMUVs are commonly detected in other domestic and wild avian species including geese^12^, layer chicken^13^, pigeons^14^, and house sparrows^3^. TMUV-infected birds exhibit various disease conditions as documented in the literature; in particular, high fever, loss of appetite, severe reduction in egg production, and/or severe neurologic disorders were commonly observed in TMUV-affected flocks^1^. Our previous study indicated that TMUV-specific antibodies and viral RNA could be detected in duck farm workers in China during the disease outbreak in ducks^15^. Although TMUV has not been reported to cause illness in humans, TMUV still has a high potential to evolve into a zoonotic pathogen that threatens public health as a mosquito-borne flavivirus. Therefore, a rapid and accurate method for diagnosing TMUV infections is important for disease control measures.

In general, TMUV is isolated using embryonated duck/chicken eggs^4^,^16^, primary duck/chicken embryo cell cultures^2^,^17^, mammalian cell lines, and mosquito cell lines^18^ for initial virology diagnosis purposes. Serological methods of virus neutralization^19^, ELISA^20^,^21^,^22^, and fluorescent antibody^23^ tests are also commonly used for the detection of TMUV antigens or specific antibodies. These TMUV routine virology and serology tests are useful, but they are time-consuming and have low-sensitivity issues. The nucleic acid amplification tests of conventional RT-PCR (cRT-PCR)^1^,^11^, nested RT-PCR, and semi-nested RT-PCR^24^ for specific TMUV genes have been effectively used for the rapid diagnosis of TMUV infection since their development during the TMUV-related disease outbreak in the late 1990s and in recent years. Furthermore, SYBR^®^ Green and TaqMan^®^ real-time RT-PCR (rRT-PCR)^25^,^26^ and reverse-transcription loop-mediated isothermal amplification (RT-LAMP)^27^,^28^ targeting specific TMUV genes were reported for more rapid and sensitive detection of TMUV infections. The advantages of RT-LAMP assays for TMUV detection in field situations or poorly equipped laboratories were previously reported by our group and other groups^24^,^29^. However, there were two main limitations of the application of RT-LAMP for TMUV detection: i) most of these developed RT-LAMP assays were based on the sequences of historical TMUV strains, and thus, they were limited to TMUV reference strains of similar origin (duck, goose, or chicken), making them unsuitable for detecting divergent TMUV strains from various hosts or genotyping groups; and ii) the high sensitivity of RT-LAMP assays can easily lead to false-positive results due to carryover contamination caused particularly by aerosol droplets generated during the assay process^30^. Fortunately, carryover contamination in the nucleotide amplification reaction could be eliminated using uracil DNA glycosylase (UDG) to process the dUTP-incorporated amplicons^31^,^32^,^33^. However, there is no report describing the integration of RNA template-based RT-LAMP amplification with UDG digestion in a one-pot reaction to eliminate carryover contaminants. Therefore, the objective of this study was to develop a carryover contamination-free rRT-LAMP assay for the rapid and universal detection of various TMUV strains.

## Results

### The rRT-LAMP primer set for the universal detection of TMUVs

**Figure 1:**
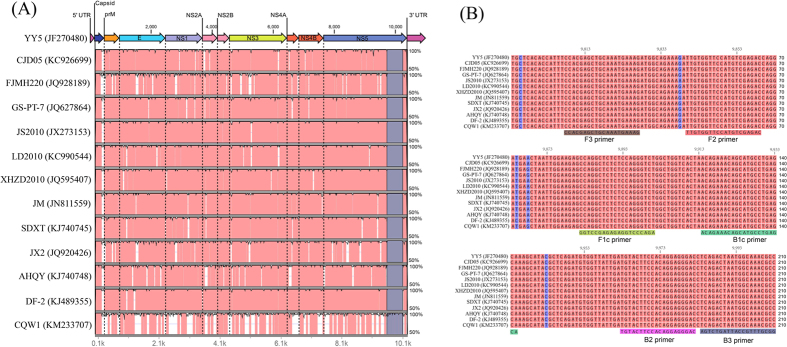
Complete open reading frame (ORF) and NS5 gene conserved region alignments of different Tembusu virus (TMUV) strains using the mVISTA method (**A**) and ClustalW method (**B**). (**A**,**B**) Illustrate the alignment results of the YY5 strain in comparisons with 12 TMUV reference strains (CJD05, FJMH220, GS-PT-7, JS2010, LD2010, XHZD2010, JM, SDXT, JX2, AHQY, DF-2, and CQW1) retrieved from GenBank (accession number is presented after the strain name). Areas in pink (**A**) exhibit ≥99% similarity and areas in white display <99% similarity. Areas in dark blue (**A**) denote the region for LAMP primer design. Areas in light blue color (**B**) represent single nucleotide differences. Six primers targeting various regions are shown according to the alignment result.

The current UDG-rRT-LAMP assay was designed to detect all TUMV genotype groups/sub-groups. We identified a conserved region among all representatives from different TMUV genotypes corresponding to the 3′ end of the NS5 gene using the mVISTA method (Fig. 1A). This conserved region was located in the RdRP domain from genome position 9,793 to 10,003 (numbering corresponds to the complete genome of strain YY5, GenBank accession No. JF270480) (Fig. 1A). The 210-bp conserved region alignment of different TMUV strains was conducted using the ClustalW method (Fig. 1B), resulting in an overall alignment score exceeding 99%. Based on the complete ORF and NS5 conserved region alignment results, four primers targeting six different regions in the NS5 gene RdRP domain (Table 1) were designed, and the six primer-targeted regions were 100% conserved among different TMUV reference strains (Fig. 1B).

### UDG-rRT-LAMP reaction

**Figure 2:**
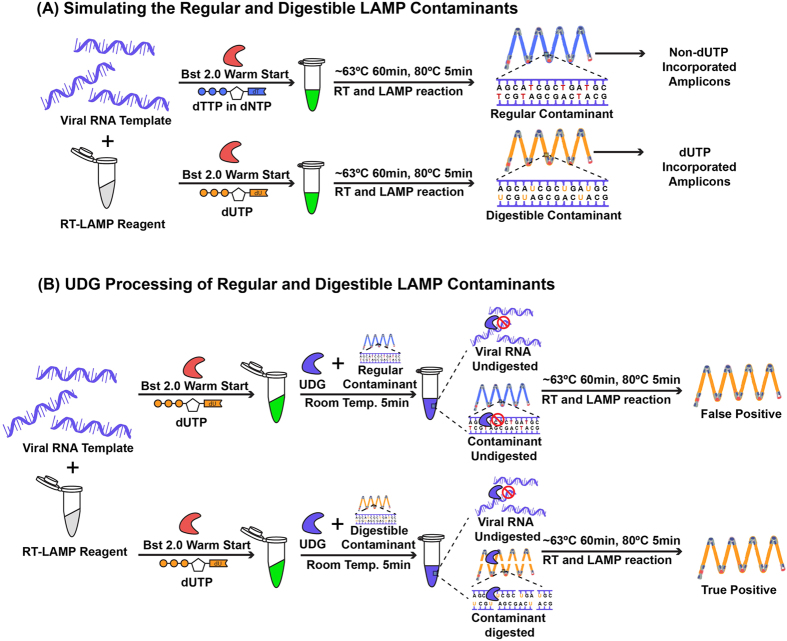
Schematic diagram showing the mechanism of the uracil DNA glycosylase-supplemented real-time reverse-transcription loop-mediated isothermal amplification (UDG-rRT-LAMP) assay. Blue LAMP amplicons represent non-dUTP-incorporated DNA and yellow LAMP amplicons represent dUTP-incorporated DNA. The red enzyme represents *Bst* 2.0 WarmStart^®^ DNA polymerase and the blue enzyme represents UDG. UDG-rRT-LAMP eliminates carryover contamination via two steps. (**A**) The first step of the incorporation of dUTP in all LAMP amplicons. (**B**) The second step of UDG-based elimination of carryover contaminants by specifically cutting the LAMP amplicon DNA at the 5′ side of the dUTP-incorporated templates from previous LAMP reactions while having no effect on non-dUTP-incorporated DNA and RNA templates. During the RT-LAMP reaction, the digested contaminants are degraded into small fragments, and UDG is inactivated at approximately 63 °C, ensuring that only the RNA template is amplified.

The developed UDG-rRT-LAMP is a one-pot, two-step assay for the specific detection of TMUV and elimination of carryover contamination. Compared with the RT-LAMP assay, our developed assay was supplemented with dUTP, which can incorporate dUTP into all RT-LAMP amplicons after amplifying the target RNA sequence under certain conditions (Table 2). Such processing was considered the first step for carryover contamination elimination (Fig. 2A). To digest the dUTP incorporated RT-LAMP products, UDG treatment was performed before isothermal amplification in step 2 (Fig. 2B), whereas the template RNA and carryover contamination of non-dUTP incorporated RT-LAMP amplicons could not be digested under this step (Fig. 2B).

### Sensitivity and specificity

**Figure 3:**
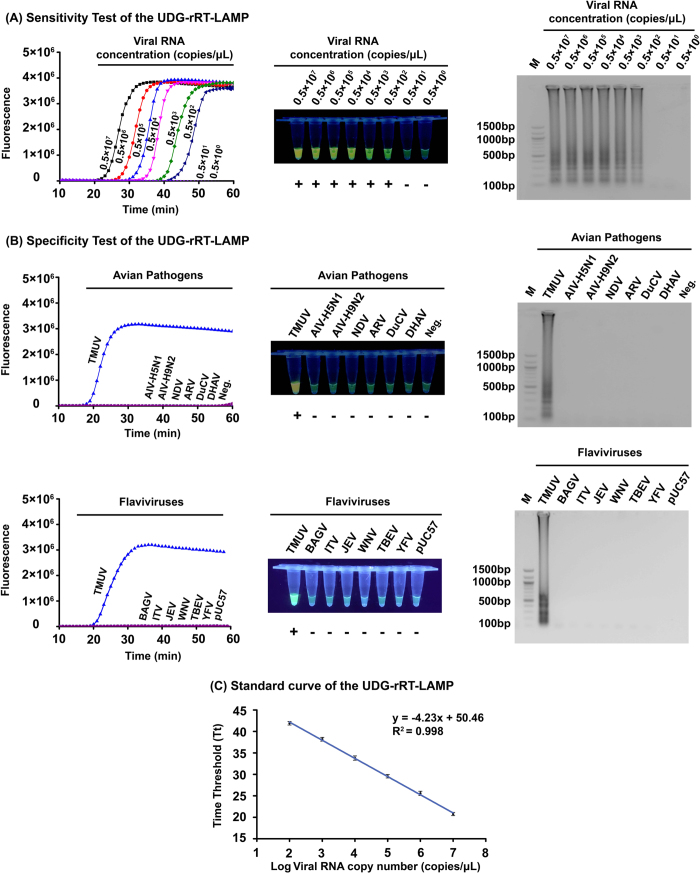
Sensitivity, specificity, and standard curve of the uracil DNA glycosylase-supplemented real-time reverse-transcription loop-mediated isothermal amplification (UDG-rRT-LAMP) assay. (**A**) Sensitivity test result of UDG-rRT-LAMP using 10-fold serial dilutions of *in vitro*-transcribed NS5 RdRP domain RNA (concentration diluted from 0.5 × 10^7^ to 0.5 × 10^0 ^copies/μL) as determined using amplification plots (left), EvaGreen^®^ fluorescent dye (middle), and agarose gel electrophoresis (right). (**B**) upper: Specificity test result of UDG-rRT-LAMP using different avian pathogens as determined using amplification plots (left), EvaGreen^®^ fluorescent dye (middle), and agarose gel electrophoresis (right); lower: Specificity test result of UDG-rRT-LAMP using partial NS5 genes of different flaviviruses as determined using amplification plots (left), EvaGreen® fluorescent dye (middle), and agarose gel electrophoresis (right). (**C**) Standard curves showing amplification of successive 10-fold dilutions of *in vitro*-transcribed NS5 RdRP domain RNA (from 0.5 × 10^7^ to 0.5 × 10^2 ^copies/μL). The Tt values in the plot were the means of three independent experiments. Error bars indicate standard deviation.

The sensitivity of the UDG-rRT-LAMP assay was studied by testing each of the 10-fold serial dilutions of the transcribed NS5 RdRP domain RNA of the TMUV-SD2010 strain and extracted RNA from 10-fold serial dilutions of titrated TMUV viruses. As a result, the UDG-rRT-LAMP assay for the universal detection of TMUVs detected a minimal of 100 copies of transcribed NS5 gene RNA per reaction (Fig. 3A) and 1 × 10^0.89^ − 1 × 10^1.55^ TCID_50_ of different Shandong TUMV field variant strains from different hosts (Table 3). The detection limit of the cRT-PCR for TMUV was determined on the same viral RNA samples in UDG-rRT-LAMP tests for comparison between the two assays. The detection limits of cRT-PCR were 1 × 10^2.17^ −1 × 10^4.55^ TCID50, indicating that the assay was 10–1000-fold less sensitive than UDG-rRT-LAMP. In addition, triplicate sensitivity tests of UDG-rRT-LAMP were performed, and the average Tt values of different dilutions were used to generate the standard curve plots (Fig. 3C). An extremely close linear correlation was observed between the Log of the NS5 gene copy numbers and the Tt values (R^2^ = 0.998), with a regression line revealing an average intercept of 50.46 ± 0.32 and an average slope of −4.23 ± 0.06.

To determine the specificity of UDG-rRT-LAMP in detecting TMUV, six other avian pathogens and negative control samples (allantoic fluid from SPF duck embryo) were included in specificity tests. No amplification signal was observed with avian influenza virus (AIV) subtypes H5N1 and H9N2, Newcastle disease virus (NDV), avian orthoreovirus (ARV), duck circovirus (DuCV), duck hepatitis A virus (DHAV), or the negative control (Fig. 3B, upper), or the negative control (Fig. 3B, upper). To further confirm the developed assay have no cross-reactions with non-TMUV flaviviruses, the synthesized last 1500 bp NS5 gene of Bagaza virus (BAGV), Israel turkey meningoencephalomyelitis virus (ITV), Japanese encephalitis virus (JEV), West Nile virus (WNV), tick-borne encephalitis virus (TBEV), and yellow fever virus (YFV) in pUC57 vector were also included in specificity test and no amplification signal was observed with these simulated viral nucleic acid samples (Fig. 3B, lower). Above results indicated that the developed UDG-rRT-LAMP assay has high specificity.

### Reproducibility

To evaluate the reproducibility of the developed UDG-rRT-LAMP method, samples containing concentrations of transcribed NS5 gene RNA of the TMUV-SD2010 strain ranging from 0.5 × 10^2^ to 0.5 × 10^7 ^copies/μL were tested in replicates of three in the same run (intra-assay) and individually in three independent runs (inter-assay) (Table 4). In both intra- and inter-assay analyses, all of the samples yielded a positive result, and the proportion of each dilution yielding a positive result was 1.00. All triplicate amplifications of each dilution resulted in minor differences in Tt values but the standard deviation did not exceed 0.80 for any replicate. The coefficients of variation for the Tt values determined for each viral RNA concentration varied from 0.22 to 1.08% in the intra-assay analysis and from 1.06 to 3.33% in the inter-assay analysis (Table 4).

### rRT-LAMP and UDG-rRT-LAMP detect simulated carryover contamination

**Figure 4:**
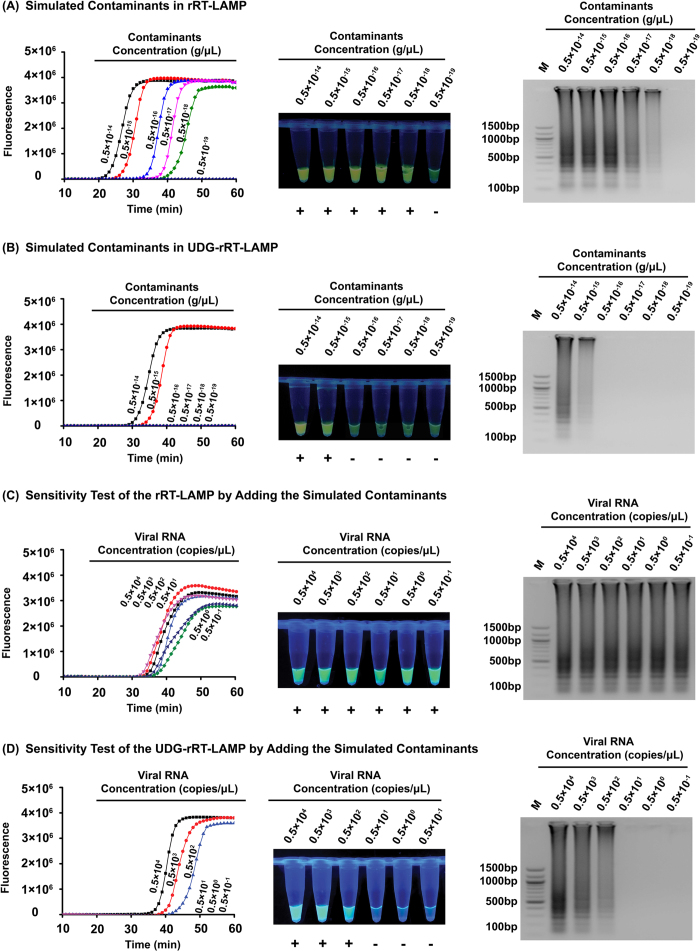
Control of carryover contamination in the uracil DNA glycosylase-supplemented real-time reverse-transcription loop-mediated isothermal amplification (UDG-rRT-LAMP) assay. Sensitivity test result of rRT-LAMP (**A**) and UDG-rRT-LAMP (**B**) using 10-fold serial dilutions of simulated carryover contamination (dUTP-incorporated LAMP amplicons DNA, concentration diluted from 0.5 × 10^−14^ to 0.5 × 10^−19 ^g/μL) as determined using amplification plots (left), EvaGreen^®^ fluorescent dye (middle), and agarose gel electrophoresis (right). Sensitivity test result of rRT-LAMP (**C**) and UDG-rRT-LAMP (D) using 10-fold serial dilutions of *in vitro*-transcribed NS5 RdRP domain RNA (concentration diluted from 0.5 × 10^−4^ to 0.5 × 10^−1 ^copies/μL) and 1 × 10^−18 ^g/reaction of simulated carryover contamination as determined using amplification plots (left), EvaGreen^®^ fluorescent dye (middle), and agarose gel electrophoresis (right).

To confirm that simulated carryover contamination from dUTP-incorporated RT-LAMP amplicons is sufficient to contaminate new reactions, we compared the detection limits of the rRT-LAMP and UDG-rRT-LAMP methods using serial diluted rRT-LAMP amplicons with concentrations ranging from 0.5 × 10^−19^ to 0.5 × 10^−14 ^g/μL. In the absence of UDG treatment, rRT-LAMP can detect as little as 1 × 10^−18 ^g of simulated carryover contamination per reaction (Fig. 4A), which is equivalent to an aerosol droplet diameter size of approximately 0.124 μm. This result indicated that even at extremely low concentrations or small particle sizes, carryover contamination can cause false positive results. With UDG treatment, the UDG-rRT-LAMP assay could only detect 1 × 10^−15 ^g or more of simulated carryover contamination per reaction (Fig. 4B), equivalent to an aerosol droplet diameter size of approximately 1.241 μm. These results demonstrated that UDG prevented the amplification of up to 1000-fold higher concentrations of carryover contaminant DNA and 1000-fold smaller sizes of aerosol droplets generated by UDG-rRT-LAMP products which significantly reduce the likelihood of false-positive results of TMUV diagnosis. A UDG concentration of 0.01 U/μL in the UDG-rRT-LAMP reaction mixture is sufficient to eliminate as much as 1 × 10^−16 ^g of dUTP-incorporated carryover contamination per reaction.

### UDG-rRT-LAMP can avoid false-positive results

To confirm that UDG-rRT-LAMP can reduce the likelihood of false-positive results due to carryover contaminants, we performed sensitivity testing of rRT-LAMP and UDG-rRT-LAMP reactions with 10-fold serial dilutions of the transcribed NS5 gene and added 1 × 10^−18 ^g of simulated carryover contamination to each reaction tube. Without UDG treatment, we could not determine the detection limit of the assay, and all tested samples displayed positive results (Tt value of 30.8–37.4), including samples with undetectable levels of viral RNA (less than 0.5 × 10^2 ^copies/μL) (Fig. 4C). These results were considered false positive. With UDG treatment, the detection limit of the rRT-LAMP assay (0.5 × 10^2 ^copies/μL) (Fig. 4D) was consistent with the aforementioned sensitivity study. Such results were considered positive. From these data, the developed UDG-rRT-LAMP assay could successfully eliminate carryover contamination.

### UDG-rRT-LAMP detects TMUV in clinical samples

To assess whether the developed TMUV UDG-rRT-LAMP assay allows the detection of viral RNA in clinical samples, a total of 81 samples were analyzed by UDG-rRT-LAMP and VI combined with NS5 gene-based cRT-PCR. Of these, 62 samples were positive according to both UDG-rRT-LAMP and VI, 17 samples were negative according to both assays, 2 samples were positive according to UDG-rRT-LAMP and negative according to VI, and 0 samples were negative according to UDG-rRT-LAMP and positive according to VI. The results of the two methods are summarized in Table 5 for comparison. Statistical analysis illustrated that the two methods have 96.88% agreement and the difference between the two methods was not significant (P > 0.05) in detecting clinical samples. Samples from non-inoculated SPF duck embryos tested negative using the TMUV UDG-rRT-LAMP assay in the same run described previously.

## Discussion

TMUV was originally identified as a tropical pathogen in Malaysia during the 1970s, and the main hosts of the virus were confirmed to be different bird species, which were responsible for the maintenance and transmission of TMUV in nature^34^. Severe diseases with mortality caused by the infection of birds with TMUV were indicated for the first time in chickens during the 1990s^11^ and subsequently studied in ducks in the early 2010s^1^,^2^. Our recent study reported a potential case of human infection with TMUV^15^, highlighting the urgent need for effective TMUV infection control and prevention methods, particularly detection methods. Several studies reported molecular methods for the detection of TMUV^1^,^11^,^24^,^25^,^26^. Nevertheless, those PCR-based assays require significant time, multiple steps, expensive instruments, and well-trained personnel to perform the experiments^24^. The use of LAMP technology is increasing in popularity for detection of TMUV and other viruses^27^,^28^ because of its rapid, simple, sensitive, specific, and isothermal reaction capacity without the need for a specialized thermal cycler. In this study, we applied an advanced UDG-rRT-LAMP method to the detection of all recognized TMUV-related viruses.

Unlike RT-PCR or rRT-PCR, which requires one pair of primers or one pair of primers and a probe, RT-LAMP requires at least four primers that recognize six regions of the target RNA sequence, and thus, its specificity is extremely high^30^. On the contrary, to achieve high specificity, the LAMP primers must have a strict match with their target sequences, or failure of amplification will occur^35^. Thus, to develop a universal TMUV detection assay, identifying conserved regions in the TMUV genome sequence is a critical step in RT-LAMP primer design. Recently, Dai *et al*.^36^ provided evidence of natural recombination in TMUV and genetic divergence between various TMUV strains isolated in different years. Therefore, in the present study, we selected the complete ORFs of 13 representative TMUV reference strains from genotyping groups 1 and 2 (including subgroups 1–3) to screen highly conserved regions among these strains for UDG-rRT-LAMP assay development. Based on the visible alignment result, we found few regions in the TMUV ORFs that were sufficiently conserved for designing the LAMP primers for the universal detection of different TMUV strains. A TMUV variant, CQW1 (KM233707), was identified to be extremely different from other reference strains concerning its E, NS3, and NS5 genes, which were commonly used in detection assay development in previous studies^1^,^29^,^37^. Fortunately, we identified a 210-nt candidate region with relatively high sequence conservation in the 3′-terminus of the NS5 gene, which corresponds to the RdRP domain with >99% nucleotide identity. The size of this region was sufficient to meet the requirement of LAMP primer set designing. The highly conserved features of this region distinguished our NS5 RdRP domain-based UDG-rRT-LAMP assay from other published assays.

Although LAMP has been revealed to be a highly sensitive and specific assay in the clinical diagnosis of many pathogens, it was also found to be highly susceptible to carryover contamination due to its high sensitivity^32^. The LAMP result is commonly confirmed by DNA electrophoresis and direct observation after adding a fluorescent dye. In these processes, both observation methods require the opening of reaction tubes, easily generating aerosol droplets of different sizes that contain high concentrations of amplicons^38^. Our present study revealed that an extremely small amount of amplification products (1 × 10^−18 ^g/reaction) from previous LAMP reactions could serve as carryover contamination templates for re-amplification, resulting in false-positive results. When converting the minimum contaminants dose that can cause false-positive results to the size of aerosol droplets, the particle diameter was found to be extremely small (approximately 0.124 μm). This could not be filtered by most routine processing methods in the laboratory. Importantly, due to the current lack of an effective strategy for eliminating LAMP carryover contamination, preventing carryover contamination relies solely on careful operation of the LAMP experiment. Once contamination occurs in the laboratory, the procedure for eliminating or reducing contamination is usually complicated, costly, time-consuming, and sometimes requires moving the experiment to a new laboratory room or designing a set of primes targeting different genomic regions^32^.

Although there were some pre- and post-amplification strategies for preventing or eliminating carryover contamination in other nucleic acid amplification assays, including mechanical/chemical barriers^38^, UV light irradiation^39^, primer hydrolysis^40^, and hydroxylamine treatment^41^, these methods may not be able to completely eliminate or prevent carryover contamination. Therefore, they are not ideal approaches for controlling carryover contamination in LAMP reaction^33^. Compared with the aforementioned methods, the UDG-mediated digestion of dUTP-incorporated contaminant DNA is simple, inexpensive, and does not require substantial modification of existing protocols^42^. It specifically removes dUTP bases in uracil-containing DNA but has no effect on natural DNA^43^ and RNA templates^44^. The prevention of carryover contamination using dUTP and UDG has been well documented in previously reported PCR and LAMP assays using both DNA and RNA as the template^31^,^32^,^33^,^45^. For RNA samples used in UDG-LAMP reactions, reverse transcription, UDG treatment, and the LAMP reaction are usually performed in separate steps^33^, which necessitates opening the reaction tube and increasing the risk of exposing the reaction mixture to carryover contaminants in the environment. Such a disadvantage limits the practical application of the UDG-RT-LAMP assay in the clinical detection of RNA viruses. Therefore, we performed rRT-LAMP amplification with UDG digestion in a one-pot reaction to eliminate carryover contaminants for the first time. The developed UDG-rRT-LAMP assay could remove as much as 1 × 10^−16 ^g/reaction of simulated contaminants with a UDG concentration of only 0.01 U/μL. Although the simulated contaminants cannot be removed at high concentrations (1 × 10^−15 ^g/reaction or more), adding more UDG and physically separating large aerosol droplet particles could serve as backup methods. To avoid opening the reaction tube during result detection, EvaGreen^®^ fluorescent dye was integrated into the RT-LAMP reaction for quantitative monitoring of the amplification and observing the endpoint result visually. Moreover, *Bst* 2.0 warmStart^®^ DNA polymerase was used in our strategy to enhance dUTP incorporation, amplification speed, amplicon yield, salt tolerance, thermostability, and specificity in the assay, which are critical to the success of the UDG-rRT-LAMP assay for TMUV detection.

In conclusion, based on the complete ORF alignment of various TMUV strains from different genotyping groups, we developed a universal TMUV detection assay using rRT-LAMP combined with UDG treatment to prevent/eliminate carryover contamination. This UDG-rRT-LAMP method is a rapid, sensitive, specific, and reliable assay for diagnosing TMUV infection, and it provides a potentially powerful strategy for avoiding false-positive results in detecting other flaviviruses.

## Materials and Methods

### Ethics statement

Viral propagation and testing were performed according to the approved the Institutional Biosafety Committee, Shandong Agricultural University. All tissue samples analyzed in this study were collected for diagnostic purposes and approved by Animal Welfare Committee, Shandong Agricultural University (License number: 2015-Vet-012).The animal care and research were conducted according to *Guide for the Care and Use of Laboratory Animals, National Research Council, 2011* (https://grants.nih.gov/grants/olaw/Guide-for-the-Care-and-use-of-laboratory-animals.pdf).

### Primer design

To identify the most conserved region among 10 genes from different TMUV strains, 13 complete ORFs of TMUV strains (isolated from 2010 to 2014) were used in this study, including 11 duck, 1 layer, and 1 goose strain. These strains represent all different TMUV genotyping groups/subgroups described in a recent TMUV evolutionary analysis study^36^. The alignment of complete ORFs was conducted using the mVISTA online program (http://genome.lbl.gov/vista/mvista/submit.shtml) and the pairwise comparison of the selected conserved region was performed using the ClustalW method in Genomic Workbench V7.5 software (QIAGEN, Boston, MA, USA). Since the most highly conserved regions were located at the 3′ end of the NS5 gene among different TMUV strains, the last 210 bp of the NS5 gene were chosen as the optimal target region for RT-LAMP primer sequence basis. Primers were designed and optimized for sequence conservation using an online program (PrimerExplorer V4, http://primerexplorer.jp/elamp4.0.0/index.html). The designed primer set contains four primers: F3, Forward Inner Primer (FIP), Backward Inner Primer (BIP), and B3. FIP (BIP) consists of the sequence of the F1c (B1c) and F2 (B2) regions. The specificity of the primers was further confirmed using a BLAST search in the NCBI nucleotide database.

### Virus strains, cell lines, and RNA extraction

For the UDG-rRT-LAMP evaluation test, six TUMV field variant strains isolated from ducks, ducklings, geese, house sparrows, and mosquitoes in Shandong Province were propagated in a African green monkey kidney cell line (Vero) (CCL-81, ATCC, Manassas, VA, USA), and titrated in tissue culture infectious doses (TCID50). AIV-H5N1, AIV-H9N2, NDV, ARV, DuCV, DHAV were also included in this study for specificity testing. Viral RNA was directly extracted from a 20% suspension (w/v) of viral transport medium diluted clinical samples, cell culture supernatant, and allantoic fluid using an RNeasy Mini Kit (QIAGEN, Valencia, CA, USA) following the manufacturer’s instructions. Briefly, 250 μL of sample was mixed with same volume of lysis buffer and then applied to the RNA binding column. After three washes, the contaminants were efficiently washed away, and total RNA eluted in 50 μL of RNase-free water was used for further testing.

### *In vitro* transcription of the partial NS5 gene

The *in vitro*-transcribed NS5 RdRP domain RNA of a TMUV field strain isolated from Peking duck (TMUV-SD2010)^4^ was used to determine the detection limit of the assay. The NS5 RdRP domain (1084 bp) of TMUV-SD2010 was amplified using published procedures^46^ and cloned into the pGEM-T easy Vector system (Promega, Madison, WI, USA) as described in our previous study^4^. The recombinant plasmid pGEM-NS5 was linearized by *Eco*RI (Promega), extracted using phenol–chloroform–isoamyl alcohol (25:24:1), precipitated using ethanol, and suspended in water before using the DNA for *in vitro* transcription reactions. The production of “run-off” transcripts derived from the inserted M1 segment used the Riboprobe^®^ *in vitro* Transcription System with T7 RNA Polymerase (Promega) following the manufacturer’s instructions. The transcribed RNA concentration was quantitated using a NanoDrop™ 1000 spectrophotometer (Thermo Scientific, Waltham, MA, USA) and copy numbers were calculated as previously described^47^ before making a 10-fold serial dilution for sensitivity testing and generating the standard curve.

### UDG-rRT-LAMP conditions

The UDG-rRT-LAMP for the NS5 RdRP domain was conducted in a 20-μL reaction system. The total reaction master mix volume was 18 μL consisting of 1.6 μM each of FIP and BIP primers, 0.2 μM each of F3 and B3 primers, 1.6 mM dNTPs (Invitrogen, Waltham, MA, USA), 1 M betaine (Sigma-Aldrich, St. Louis, MO, USA), 4 mM MgSO_4_ (New England Biolabs, MA, USA), 5 U of AMV reverse transcriptase (Promega), 100 mM dUTP (New England Biolabs), 0.2 U UDG (New England Biolabs), 1× ThermoPol reaction buffer, 8 U of *Bst* 2.0 warmStart^®^ DNA polymerase (New England Biolabs), and 1× EvaGreen^®^ fluorescent dye (Biotium, Hayward, CA, USA). After adding 2 μL of viral RNA, the reaction mixture was incubated at room temperature for 5 min to eliminate carryover contamination. Following incubation, the reaction was performed using the 7500 Real-time PCR System (Applied Biosystems, Foster City, CA, USA) for rRT-LAMP or with a water bath for RT-LAMP at 63 °C for 1 h. The thermal cycling profile for rRT-LAMP proceeded as follows: 60 cycles of 62.5 °C for 5 s and 63.5 °C for 55 s. Fluorescence signals for each sample were collected at the end of the 63.5 °C step. The time threshold (Tt) values and standard curve were analyzed using the SDS software program (version 1.4) and visualized using OriginPro 8.5 software (OriginLab, Northampton, MA, USA). The rRT-LAMP results were also detected by fluorescence observation under UV light and 2% (w/v) agarose gel electrophoresis.

### Sensitivity, specificity, and reproducibility of UDG-rRT-LAMP

The sensitivity of the UDG-rRT-LAMP assay was determined in two separate systems. One system was transcribed NS5 RdRP domain RNA of the TMUV-SD2010 strain, which was diluted from 0.5 × 10^7^ to 0.5 × 10^0 ^copies/μL. The other system was extracted RNA from 10-fold serial dilutions of titrated TMUV field variant strains isolated from different hosts in Shandong province. Three replicates for each dilution of different samples were tested using the developed UDG-rRT-LAMP assay. The detection limit of the assay was determined as the last dilution at which all three replicates from each dilution gave a positive result. Samples of serial dilutions of six TUMV field variant strains were also tested by NS5 gene-based cRT-PCR^46^ to compare its sensitivity with that of UDG-rRT-LAMP.

The specificity of UDG-rRT-LAMP was first examined using viral RNA from different avian pathogens of AIV-H5N1, AIV-H9N2, NDV, ARV, DuCV, and DHAV to confirm that no cross-reactions with non-targeted viral RNA occurred. The allantoic fluid from SPF duck embryos was also included in the test as a negative control. To further confirm the developed assay have no cross-reactions with non-TMUV flaviviruses, the last 1500 bp NS5 gene sequences of BAGV (GenBank accession no. EU684972), ITV (GenBank accession no. KC734549), JEV (GenBank accession no. AF080251), WNV (GenBank accession no. AY532665), TBEV (GenBank accession no. FJ402886) and YFV (GenBank accession no. U54798) were synthesized and cloned into pUC57 vector (Genscript, Nanjing, Jiangsu, China) for specificity test purpose and the empty pUC57 vector was working as a negative control.

The reproducibility of the developed assay was assessed using transcribed NS5 RdRP domain RNA from the TMUV-SD2010 strain. The RNA was diluted from 0.5 × 10^7^ to 0.5 × 10^2 ^copies/μL to prepare the intra- and inter-assay samples. Replicate samples of each dilution were stored at −80 °C immediately after preparation. For the intra-assay test, three replicate samples from each dilution were tested in the same run. For the inter-assay test, samples from different dilutions were tested in three independent runs. Reproducibility was measured by calculating the proportion of positive samples and determining Tt values of each sample. The coefficient of variation was determined to find statistical correlations.

### Simulating carryover contamination

The amplicons of the RT-LAMP reaction generated from 0.5 × 10^4 ^copies/μL of transcribed NS5 RdRP domain RNA of the TMUV-SD2010 strain in the absence of UDG, which served as the source of simulating carryover contaminants, was quantitated using the NanoDrop™1000 spectrophotometer to generate a 10-fold serial dilution from 0.5 × 10^−14^ to 0.5 × 10^−19 ^g/μL. When using 0.2 μL of diluted RT-LAMP amplicons as the template in the UDG-rRT-LAMP assay, the total mass of carryover contaminants was approximately 1 × 10^−19^ − 1 × 10^−14^ g/reaction (equivalent to aerosol droplet diameter size of 2.673 μm,1.241 μm, 0.567 μm, 0.267 μm, 0.124 μm and 0.057 μm, respectively).

### Elimination of carryover contamination by UDG-rRT-LAMP

To evaluate the ability of the developed UDG-rRT-LAMP method to reduce false-positive results due to carryover contaminants in detecting TMUV, we conducted both rRT-LAMP and UDG-rRT-LAMP reactions by adding 2 μL of diluted viral RNA templates (0.5 × 10^−1^ − 0.5 × 10^4 ^copies/μL) and 2 μL of simulated carryover contamination of 0.5 × 10^−18 ^g/μL in the same reaction tube. The total mass of the simulated carryover contamination for each reaction was approximately equivalent to a 0.124 μm-diameter aerosol droplet, which cannot be efficiently blocked by either high efficiency particulate air (HEPA) filters^48^ in the biosafety cabinets or fibrous pipette tip filters^49^. The detection limits of the rRT-LAMP reactions with and without UDG treatment before amplification were compared to confirm whether the studied UDG-rRT-LAMP assay can eliminate false positives.

### UDG-rRT-LAMP detect TMUV in clinical sample

Eighty-one original tissue specimens, including theca folliculi, oviduct, heart, and liver from ducks or other avian species that exhibited clinical signs and lesions consistent with those of TMUV infections during necropsies, were used to evaluate UDG-rRT-LAMP in detection TMUV from clinical poultry specimens. The collected tissue samples were minced using scissors in viral transport medium^50^ to produce a 20% suspension (w/v). The tissue suspension was further processed in a stomacher blender (Model 80, Seward Ltd., Davie, FL, USA) and centrifuged at 1200 rpm for 10 min at 4 °C. Thereafter, the processing tissue supernatant was filtered through a 0.45-nm syringe filter for UDG-rRT-LAMP detection and virus isolation (VI) in Vero cells. TMUV-positive isolates were confirmed by NS5 gene-based cRT-PCR^46^ to detect TMUV-infected cytopathic effect cells from the specimen-inoculated Vero cell cultures. Lastly, the agreement between UDG-rRT-LAMP and VI results was summarized to investigate the clinical performance of our rRT-PCR assays. Statistically significant differences between the two assays were determined using the two-tailed Student’s *t*-test in the Microsoft Excel program.

## Additional Information

**How to cite this article**: Tang, Y. *et al*. Advanced uracil DNA glycosylase-supplemented real-time reverse transcription loop-mediated isothermal amplification (UDG-rRT-LAMP) method for universal and specific detection of Tembusu virus. *Sci. Rep.* **6**, 27605; doi: 10.1038/srep27605 (2016).

## Figures and Tables

**Table 1:** Tembusu virus (TMUV) real-time RT-LAMP (rRT-LAMP) primers and conventional RT-PCR (cRT-PCR) for the NS5 gene used in this study.

**Assay**	**Oligo**	**Sequence (5′–3′)**	**Length (bp)**	**Positions (Genome)**^2^
rRT-LAMP for NS5 gene^1^	F3 primer	CCACGAGCTGCAAATGAAAG	20	9,808–9,827
	B3 primer	GGCGTTTGCCATTAGTCTGA	20	9,984–1,0003
	FIP primer	AGACCCTGGAGAGAGCCTGG-	40	9,882–9,901
	(F2 + F1c)	TTGTGGTTCCATGTCGAGAC		9840–9,859
	BIP primer	ACAGAAACAGCATGCCTGAGCA-	42	9,914–9,935
	(B2 + B1c)	GTCCCTCCTGTGGAAGTACA		9,963–9,982
cRT-PCR for NS5 gene^3^	FU1b	TACAACATGATGGGVAARAGWGARAA	26	9,014–9,039
	cFD3b	ARCATGTCTTCYGTBGTCATCCA	23	10,076–10,098

^1^The rRT-LAMP for the NS5 gene primers and probe were newly designed in this study.

^2^Number correspond to the first nucleotide position in the genome of TMUV YY5 strain (Genbank accession number: JF270480).

^3^The cRT-PCR for the NS5 gene primers were originally designed by Kuno^35^.

**Table 2:** Tembusu virus (TMUV) uracil-DNA-glycosylase real-time RT-LAMP (UDG-rRT-LAMP) reaction components.

**Component**	**Final Concentration**	**Stock Concentration**	**Volume**
Tris-HCl	20 mM	10× reaction buffer	2 μL
(NH_4_)_2_SO_4_	10 mM		
KCl	50 mM		
MgSO_4_	2 mM		
Tween-20	0.1%		
MgSO_4_	6 mM	100 mM	1.2 μL
dNTP	1.4 mM	10 mM	2.8 μL
dUTP	1.4 mM	100 mM	0.28 μL
FIP	1.6 μM	10× primer mixture	2 μL
BIP	1.6 μM		
F3	0.2 μM		
B3	0.2 μM		
Bst2.0	0.32U/μL	8U/μL	0.8 μL
EvaGreen dye	1×	20×	1 μL
MuLV	20U/μL	200U/μL	1 μL
Cod-UDG	0.01U/μL	1U/μL	0.2 μL
H_2_O			6.72
RNA sample			2 μL
Total volume			**20 μL**

**Table 3:** Detection limitations for Tembusu virus (TMUV) uracil-DNA-glycosylase real-time RT-LAMP (UDG-rRT-LAMP) compared with conventional RT-PCR (cRT-PCR).

TMUV field strain	Host	Isolated Year	Virus titer^1^	**Detection titer limit**	
				UDG-rRT-LAMP	cRT-PCR
TMUV-SD2010	Duck	2010	10^6.89^	10^0.89^	10^3.89^
TMUV-SDXT	Duck	2011	10^6.12^	10^1.12^	10^3.12^
TMUV-SDLZ	Duckling	2011	10^6.55^	10^1.55^	10^4.55^
TMUV-SDZC-1	Goose	2011	10^7.32^	10^1.32^	10^3.32^
TMUV-SDHS	Sparrow	2012	10^5.33^	10^1.33^	10^3.33^
TMUV-SDMS	Mosquito	2012	10^5.17^	10^1.17^	10^2.17^

^1^The virus titer was measured in tissue culture infections dose 50% per 100 μL (TCID_50_/100 μL).

**Table 4:** Intra- and inter-assay variability of Tt values from Tembusu virus (TMUV) uracil-DNA-glycosylase real-time RT-LAMP (UDG-rRT-LAMP) detecting RNA transcripts from the TMUV-SD2010 NS5 gene.

RNA transcripts (RNA copy numbers)	**Intra-assay variability (n = 3) of CT-value**			**Inter-assay variability (n = 3) of CT-value**		
	**Mean**	**SD**	**CV (%)**	**Mean**	**SD**	**CV (%)**
10^7^	20.42	0.15	0.74	21.37	0.71	3.33
10^6^	25.25	0.27	1.08	26.10	0.49	1.86
10^5^	29.24	0.11	0.37	30.07	0.69	2.29
10^4^	33.47	0.19	0.56	33.85	0.54	1.59
10^3^	38.53	0.09	0.22	38.05	0.40	1.06
10^2^	41.57	0.11	0.26	42.14	0.56	1.32

Note: CV = coefficient variation; SD = standard deviation.

**Table 5:** List of Tembusu virus (TMUV) uracil-DNA-glycosylase real-time RT-LAMP (UDG-rRT-LAMP) and virus isolation (VI) results for clinical samples test.

**Result by**		
**UDG-rRT-LAMP**	**VI/cRT-PCR**^1^	**No. of samples (total,81)**
Pos.	Pos.	62
Neg.	Neg.	17
Pos.	Neg.	2
Neg.	Pos.	0

^1^cRT-PCR: NS5 gene-based conventional RT-PCR, described in Table 1.
